# Systemic Effects Reflected in Specific Biomarker Patterns Are Instrumental for the Paradigm Change in Prostate Cancer Management: A Strategic Paper

**DOI:** 10.3390/cancers14030675

**Published:** 2022-01-28

**Authors:** Olga Golubnitschaja, Peter Kubatka, Alena Mazurakova, Marek Samec, Abdullah Alajati, Frank A. Giordano, Vincenzo Costigliola, Jörg Ellinger, Manuel Ritter

**Affiliations:** 1 Predictive, Preventive and Personalised (3P) Medicine, Department of Radiation Oncology, University Hospital Bonn, Rheinische Friedrich-Wilhelms-Universität Bonn, 53127 Bonn, Germany; 2 European Association for Predictive, Preventive and Personalised Medicine (EPMA), 1160 Brussels, Belgium; peter.kubatka@uniba.sk (P.K.); vincenzo@emanet.org (V.C.); 3 Department of Medical Biology, Jessenius Faculty of Medicine, Comenius University in Bratislava, 03601 Martin, Slovakia; 4 Department of Obstetrics and Gynecology, Jessenius Faculty of Medicine, Comenius University in Bratislava, 03601 Martin, Slovakia; liskova80@uniba.sk (A.M.); marek.samec@uniba.sk (M.S.); 5 Department of Urology, University Hospital Bonn, Rheinische Friedrich-Wilhelms-Universität Bonn, 53127 Bonn, Germany; abdullah.alajati@ukbonn.de (A.A.); joerg.ellinger@ukbonn.de (J.E.); manuel.ritter@ukbonn.de (M.R.); 6 Department of Radiation Oncology, University Hospital Bonn, Rheinische Friedrich-Wilhelms-Universität Bonn, 53127 Bonn, Germany; frank.giordano@ukbonn.de; 7 European Medical Association (EMA), 1160 Brussels, Belgium

**Keywords:** strategic paper, prostate cancer, malignancy, benignancy, predictive diagnostics, targeted prevention, personalisation of medical services, PPPM/3P medicine, patient stratification, individualised patient profiling, risk factors, non-modifiable, modifiable, preventable, BMI, systemic effects, blood, molecular patterns, biomarker panel, ischemia, oxidative stress, DNA damage, cell-free nucleic acids, apoptosis, ageing, mitochondriopathy, chronic inflammation, homocysteine metabolism, endothelin-1, melatonin, nitric oxide, sleep quality, immune system, microbiome, behavioural patterns, liquid biopsy, metabolomics

## Abstract

**Simple Summary:**

Prostate cancer (PCa) incidence is increasing globally. The costs of treating PCa are currently increasing more rapidly than those of any other cancer, due to an overtreatment of slowly developing disease on one hand and underestimation/therapy resistance of particularly aggressive PCa subtypes on the other hand. How to reverse this trend? PCa is a multi-factorial disease resulting from an imbalanced interplay between health risks and protective factors. Suboptimal behavioural patterns and abnormal stress reactions, amongst others, cause negative systemic effects synergistically involved in PCa development and progression. Further, systemic effects are relevant for the entire human body and, therefore, are reflected in body fluids such as blood. Blood profiles specific for PCa are, therefore, instrumental for the risk assessment in PCa predisposed individuals, targeted disease prevention and personalised treatment algorithms—altogether 3P (predictive, preventive and personalised/PPPM) medicine as a new paradigm in PCa management. This strategic article initiated by the European Association for Predictive, Preventive and Personalised Medicine (EPMA, Brussels) and European Medical Association (EMA, Brussels) and created by an international multi-professional group of experts, highlights systemic effects in PCa initiation, development and progression, demonstrates evident challenges and provides expert recommendations in the framework of 3P medicine.

**Abstract:**

Prostate cancer (PCa) is reported as the most common malignancy and second leading cause of death in America. In Europe, PCa is considered the leading type of tumour in 28 European countries. The costs of treating PCa are currently increasing more rapidly than those of any other cancer. Corresponding economic burden is enormous, due to an overtreatment of slowly developing disease on one hand and underestimation/therapy resistance of particularly aggressive PCa subtypes on the other hand. The incidence of metastatic PCa is rapidly increasing that is particularly characteristic for young adults. PCa is a systemic multi-factorial disease resulting from an imbalanced interplay between risks and protective factors. Sub-optimal behavioural patterns, abnormal stress reactions, imbalanced antioxidant defence, systemic ischemia and inflammation, mitochondriopathies, aberrant metabolic pathways, gene methylation and damage to DNA, amongst others, are synergistically involved in pathomechanisms of PCa development and progression. To this end, PCa-relevant systemic effects are reflected in liquid biopsies such as blood patterns which are instrumental for predictive diagnostics, targeted prevention and personalisation of medical services (PPPM/3P medicine) as a new paradigm in the overall PCa management. This strategic review article highlights systemic effects in prostate cancer development and progression, demonstrates evident challenges in PCa management and provides expert recommendations in the framework of 3P medicine.

## 1. Introduction

### 1.1. Prostate Cancer as a Socio-Economic Burden in Focus of the Paradigm Change in Healthcare

Prostate cancer (PCa) is reported as the most common malignancy and second leading cause of death in America. To this end, the American Cancer Society recorded 191,930 new cases and 33,330 new deaths of PCa in 2020 [1]. In Europe, PCa is considered the leading type of tumour in 28 European countries accounting for almost 21.8% of all newly diagnosed cancer patients and about 10% of cancer-related deaths [2].

The costs of treating PCa are currently increasing more rapidly than those of any other cancer type. Corresponding economic burden is enormous, due to an overtreatment of slowly developing disease on one hand and underestimation/therapy resistance of particularly aggressive PCa subtypes on the other hand. To this end, an incidence of metastatic PCa is rapidly increasing exemplified by trends observed in the USA: currently, an increase of over 40% in 2025 is prognosed. Noteworthy, rapidly increasing PCa incidence rates are characteristic for adolescents and young adults aged 15–40 years that is another contributing factor to the metastatic disease developed later on in life [3].

### 1.2. PCa Is a Systemic Multi-Factorial Disease

PCa is a systemic multi-factorial disease resulting from an imbalanced interplay between risks and protective factors. This interplay is extremely comprehensive as clearly illustrated by highly individual association between the Body Mass Index (BMI) and PCa-related mortality [4]: a disease specific association between BMI and corresponding mortality rates has been demonstrated in the UK population. A detailed comparative analysis demonstrated that for the PCa patient cohort a multi-parametric analysis is essential to correctly interpret the research data presented, in order to predict individual outcomes; in contrast, for example, poor uterus malignancy outcomes are clearly predictable by overweight and obesity [3].

### 1.3. Non-Modifiable Risk Factors of PCa

A family history of PCa is a well-established risk factor of PCa [5]. Race and ethnicity are further evident risks. For example, PCa in the male “Afro-American” sub-population has been demonstrated as biologically and genetically more aggressive compared with PCa in the male “White American” sub-population [6]. Progressing age and advanced biological age, compared with the chronological one, are both PCa risk factors facilitating immune-senescence [7,8]. To this end, immune-suppressive PCa subtypes have been demonstrated as being particularly treatment-resistant [9].

### 1.4. Modifiable PCa Risks

Preventable cases are prevalent in the PCa patient cohorts worldwide. Modifiable risks are multi-faceted including unhealthy (e.g., sedentary) life-style, inappropriate dietary habits, toxic environment, disturbed circadian rhythm and sleep disorders, imbalanced stress conditions leading to mitochondrial impairments, excessive release of reactive oxygen species (ROS) and chronic inflammation, sexually transmitted diseases and shifted microbiome profiles, collateral pathologies such as metabolic syndrome, amongst others as recently reviewed by Kucera R. and colleagues [3].

Since PCa relevant risk factors are largely known being also modifiable, an evidence-based risk assessment is instrumental for individualised disease prediction, cost-effective prevention and treatments tailored to the person. This strategic article highlights systemic effects relevant for the PCa development and progression and presents corresponding analytical tools utilising liquid biopsies that allow development of cost-effective medical services by changing the paradigm from reactive to predictive, preventive and personalised medicine in the PCa relevant research and healthcare management.

## 2. PCa Relevant Systemic Effects Reflected in Blood Patterns

### 2.1. Stress Reactions and Imbalanced Antioxidant Defence in the Pathophysiology of PCa

The stress overload and shifted oxidant versus antioxidant balance are implicated in the pathomechanisms of PCa development and progression [3].

To this end, increased levels of stress and anxiety have been demonstrated for PCa patients, whereas males taking beta blockers are less predisposed to PCa development. An interaction between PCa development and progression on one hand and, on the other hand, psychosocial environment mediated by activation of an adrenaline/ADRB2/PKA/BAD anti-apoptotic signalling pathway has been concluded. Consequently, there are evidently PCa predisposed individuals who could benefit from timely identification of the stress overload, stress reduction and pharmacological inhibition of stress-induced signalling [10].

Further, levels of 8-hydroxydeoxyguanosine (8-OHdG, an oxidised DNA-nucleoside), were demonstrated as being significantly increased (*p* < 0.0001) in leucocytes of high-risk PCa subjects versus age-matched healthy controls, whereas glutathione S-transferase (GST) and reduced glutathione (GSH) levels are significantly higher in controls [11]. Moreover, 8-OHdG and PSA levels correlate positively, while GST and PSA correlate negatively. Consequently, particularly during the early stages of disease development, stress overload and oxidative damage play a role in prostate carcinogenesis, and molecular patterns reflecting antioxidant defence are considered a valuable marker in PCa prediction and prognosis.

### 2.2. Sleep and Melatonin Patterns Related to PCa Risks and Prognosis

According to the International Agency for Research on Cancer (IARC), shift work leading to circadian disruption might be carcinogenic to humans—the conclusion made based on studies of female night shift workers and flight attendants employed for at least 10 years—both groups demonstrating increased risk of breast cancer [12]. Additionally, animal studies are supportive considering a carcinogenic effect of non-physiologic light exposure during the daily dark periods. Research on PCa risks demonstrated a potential association between short sleep duration and higher risk of fatal prostate cancer suggesting that short sleep duration may be involved in PCa carcinogenesis [13]. Consequently, the relationship between melatonin disruption and PCa risks on one hand, and on the other hand the mechanism of how melatonin inhibits PCa are intensively discussed in the context of preventing, diagnosing and treating human prostate cancer for developing corresponding personalised treatment modalities [14]. Moreover, melatonin is considered a non-invasive biomarker for predicting PCa development, since low urinary melatonin levels correlate well with PCa incidence in the population.

### 2.3. Involvement of the Immune System: Blood Patterns Indicative for PCa Prediction and Patient Stratification

Flow cytometric profiling of blood immune cell subsets was demonstrated to be instrumental for non-invasive PCa diagnostics and differentiation between low- and high-risk disease [15]. Consequently, blood tests utilising machine learning prediction models and disease specific biomarker patterns distinguishing between benign prostate disease and PCa are envisaged to have the potential to transform prostate cancer diagnostics into individualised, precise and non-invasive approaches.

### 2.4. Systemic Ischemia and Interplay between Endothelin-1 and Nitric Oxide Are Crucial for PCa Development and Progression

Systemic ischemia and associated inflammatory and oxidative processes as well as reduced antioxidant defence together play a key role in PCa pathomechanisms [3,16]. In this context, the interplay between endothelin-1 (ET) and nitric oxide (NO) as mediators of vasoconstriction and vasodilation in blood vessels, respectively, is crucial for PCa development and progression. The ET-axis evidently contributes to PCa pathophysiology: in prostate carcinoma, ET-1 levels are systemically increased and silencing ET-1 by RNAi significantly suppresses the progression and invasion of prostate cancer cells. Several signalling pathways are systemically involved including Erk1/2/Bcl-2/Caspase-3, PI3K/Akt/Caspase-3, MMP-2 and MMP-9 [17].

Nitric oxide (NO), a signalling molecule and mediator of vasodilation in blood vessels, plays a regulatory role in many organ systems being, therefore, involved in processes relevant for the tumour microenvironment and inflammation. Further, NO can inhibit activity of the androgen receptor relevant for PCa development and therapies. In contrast, low levels of NO could conversely select specifically for the castration-resistant prostate cells, creating an aggressive cancer phenotype [18].

### 2.5. Systemic Inflammation

Systemic low-grade inflammation might be potentially associated with particularly aggressive cancer subtypes [19]. Further, when investigating the potential relationship between highly sensitive CRP (hs-CRP) levels and white blood cell count (WBC), a significant increase in hs-CRP levels has been found to be clearly associated with high PCa risks. Finally, an increased systemic inflammatory score (WBC, hs-CRP) is linked to a pronounced predisposition to the metastatic PCa [3].

### 2.6. Systemic Effects of Homocysteine Metabolism Axes on PCa Development and Progression

Non-physiologic homocysteine (Hcy) levels easily detectable in blood plasma leading, for example, to hyperhomocysteinemia (HHcy) are associated with several metabolic disorders, aberrant gene methylation and damage to DNA—altogether synergistically increasing risks of carcinogenesis in affected individuals as recently reviewed [20]. Specific polymorphisms in genes involved in the Hcy metabolism axes as well as diets deficient in folate, vitamin B6 or cobalamin correlate with HHcy and cascading pathologies. Further, administration of drugs such as laxatives and immunosuppressive drugs, may lead to HHcy development and subsequent folate reduction associated with a number of associated disorders in affected individuals [20]. To this end, involved in the Hcy-axes specifically, imbalanced metabolism of vitamin B12 and folate has been associated with increased prostate cancer risk [21].

## 3. Anti-Cancer Protection Relevant for PCa Development and Progression

### 3.1. Mitochondrial Dysfunction, Excessive ROS, Oxidative Stress and the Protective Role of Phytochemicals

In eukaryotic cells, mitochondria represent the primary source of energy production obtained through respiration and oxidative phosphorylation [22]. Mitochondrial respiratory chain is the main site of ROS production [23]. ROS generated by mitochondria contribute to the stress signalling in normal cells. However, mitochondrial ROS are also associated with the initiation of mitochondrial or nuclear DNA mutations and neoplastic transformation of cells [24]. Despite the presence of powerful antioxidant systems, excessive ROS is unable to be effectively neutralised while cumulative oxidative damage decreases mitochondrial efficiency and increases ROS excess [23]. Mitochondria contain their own DNA; however, mtDNA is neither protected by packaging proteins nor by the efficient repair mechanisms. Therefore, mtDNA is associated with a higher rate of somatic mutations when compared with nuclear DNA [25]. Indeed, somatic mutations and deletions of mtDNA are observed in numerous cancer types [22,24]. Increased ROS production in prostate cancer cells results in oxidative stress and associated metabolic alterations, androgen receptor activation and mutation-induced dysfunction of mitochondria [26]. The induction of nuclear factor erythroid 2-related factor 2 (Nrf2) stimulated by increased ROS is important in the protection against oxidative damage by enhancing the activity of antioxidant enzymes (catalase, superoxide dismutase and glutathione peroxidise) [27,28]. Inactivation of antioxidant genes is also implicated in prostate cancer as demonstrated by deregulated Nrf2 [26]. In addition, activated androgen receptor signalling also promotes ROS generation [29]. Therefore, exaggerated ROS and impaired antioxidant defence mechanisms are associated with prostate cancer pathogenesis and increased risk of prostate cancer [29,30]. However, numerous phytochemicals have been implicated in the prevention of prostate cancer [26]. Actually, the incidence of prostate cancer is lowest in Asian countries, which are characterised by regular consumption of soy food that is a rich source of isoflavones [31]. Cancer chemoprevention by naturally occurring phytochemicals with potent antioxidant capacity is essential for the inhibition, delay or reverse of carcinogenesis [28,32,33,34,35,36,37,38,39,40,41,42]. Flavonoid quercetin decreased the level of lipid peroxides and H_2_O_2_ and thus inhibited prostate cancer initiation in vivo [43]. Moreover, increased dietary intake and circulating lycopene, a primary bioactive component of tomatoes, is associated with reduced risk of prostate cancer [44,45]. Apart from the role of exaggerated ROS for increased risk and initiation of prostate cancer, high levels of ROS can, on the contrary, prevent distant metastasis [46]. Moreover, the activation of the antioxidant Nrf2 pathway contributes to the development of resistance due to disrupting oxidative mediated cell death by reducing ROS levels [28]. Deregulated Nrf2 signalling is implicated in the resistance of prostate cancer cells to chemotherapy and survival in oxidative conditions [47]. Nevertheless, sequential treatment of prostate cancer cells by vitamin C and quercetin decreased the expression of Nrf2 while this finding highlights the importance of the identification of key compounds to suppress Nrf2 gene expression as a strategy to increase the sensitivity of prostate cancer cells to chemotherapy [27]. In this regard, SET domain-containing lysine methyltransferase 7 (Setd7) represents an epigenetic regulator of Nrf2 pathway in prostate cancer cells. Two phytochemicals, phenethyl-isothiocyanate and ursolic acid, induced Setd7 expression that triggers the Nrf2/anti-oxidant response element (ARE) signalling and thus decreases oxidative stress and protects DNA [48]. Moreover, Wang et al. recently demonstrated that the combination of genistein and Doxorubicin hydrochloride weakens metastasis in prostate cancer cells by amplifying oxidative damage [46]. In conclusion, phytochemicals exert potent antioxidant capacity applicable to the targeted prevention and reversing of prostate cancer development [43,44,45], while the pro-oxidant capacity could repress the progression, metastasis or resistance of prostate cancer [27,46].

### 3.2. Circadian Rhythm Disruption and the Protective Role of Melatonin

Circadian rhythm is essential for many physiological functions and biological processes; circadian rhythm is controlled by a central clock in the suprachiasmatic nucleus (SCN) of hypothalamus and peripheral clocks in other brain regions or peripheral tissues regulated by circadian genes. Several circadian genes are implicated in prostate cancer [49]. Disruption of circadian rhythm exerts a significant role in carcinogenesis and facilitates the establishment of cancer hallmarks. Indeed, long-term exposure to shift-work increases cancer risk [50]. In 2007, International Agency for Research on Cancer (IARC) classified “shift work leading to a disruption of circadian rhythm” as probably carcinogenic to humans [12,49]. A recent study described the potential association between long duration of permanent night work in combination with a long shift length or at least six consecutive nights and prostate cancer, particularly in men with aggressive prostate cancer [49]. Similarly, Papantoniou et al. demonstrated an association between night shifts and prostate cancer, especially for tumours with poor prognosis [51].

Melatonin is secreted from the pineal gland under the control of SCN and is closely associated with circadian rhythm as it reaches a peak at night and is almost undetectable during the day [52]. Melatonin is essential for various biological processes and exerts anti-oxidant and anti-inflammatory properties, immunomodulatory effects and overall anticancer efficiency [53,54,55,56,57,58,59,60,61,62]. Low levels of melatonin are associated with increased risk of cancer including prostate cancer [52,63]. Alongside night shifts [49], men with reported sleep problems had lower morning levels of the primary melatonin metabolite 6-sulfatoxymelatonin (6-STM) while men with lower levels of 6-STM are at increased risk of advanced or lethal prostate cancer [64]. However, Wang et al. recently demonstrated the capacity of melatonin to impede prostate cancer metastasis through suppressing MMP-13 and thus inhibiting migratory and invasive capacities of prostate cancer cells in vitro and in vivo [65]. Additionally, a retrospective study conducted on prostate cancer patients revealed the capacity of melatonin to increase overall survival in patients with poor prognosis after combined hormone radiation treatment [66]. Moreover, circadian science based on the optimal timing of food and light exposure can reduce the risk of prostate cancer [50]. Therefore, disrupted circadian rhythm and associated altered release of melatonin are suggested to contribute to prostate cancer pathogenesis [49,51,64], while current research suggests the potential ability of an increased level of melatonin to repress the progression of prostate cancer [65,66]. Although substantial evidence (mainly preclinical) points to fact that melatonin could be a novel clinical strategy in the management of prostate cancer, further in-depth evaluations of the melatonin-induced oncostatic effects on prostate cancer are worthy of more attention. More efforts are needed to translate current non-clinical research of melatonin to clinic practice [14].

### 3.3. Inflammation and the Protective Role of Anti-Inflammatory Substances

Inflammation is defined as a physiological process resulting from the exposure to various infections or tissue injuries. However, persistent inflammation is associated with the production of ROS, reactive nitrogen species, chemokines, cytokines and growth factors that, when produced persistently, potentially result in uncontrolled proliferation, cellular and genomic instability, thus increasing the risk of cancer [67]. Growing evidence supports the role of inflammation in both development and progression of prostate cancer [68,69]. The significant effects of chronic inflammation on prostate cancer is mediated through affecting the tumour microenvironment, immune responses, angiogenesis, epithelial-mesenchymal transition (EMT), migration and metastasis [68,69,70,71]. Indeed, Gurel et al. demonstrated common chronic inflammation in benign prostate tissue and also an association between chronic inflammation and odds of prostate cancer, in particular high-grade. These results suggest the need to identify individuals at higher risk as a first step to prevent lethal prostate cancer [68].

Naturally occurring phytochemicals are considered as important anti-cancer substances targeting various signalling cascades deregulated in cancer including inflammation [32,37,70,72,73,74,75,76,77]. Actually, dietary lycopene, a primary bioactive component of tomatoes, has been associated with reduced risk of prostate cancer while a reduction in lycopene levels correlate with prostate cancer incidence [78]. Indeed, lycopene exerts significant capacity to inhibit prostate cancer progression demonstrated by reduced levels of inflammatory factors including interleukin (IL)-1, IL-6, Il-8 and tumour necrosis factor-α (TNF-α) in prostate cancer cells in vitro. Moreover, the increasing dose of lycopene led to improved survival of prostate cancer bearing xenografts [78]. Moreover, a case-control study describes an 83% reduction in prostate cancer risk in the individuals with the highest plasma concentration of lycopene [79,80]. However, the association between protective effects of lycopene/tomato products and prostate cancer have not been confirmed by other studies [81,82]; nevertheless, the evaluation of the effects of lycopene in individuals with a history of prostate cancer requires additional analysis [81]. With exception of lycopene, there are also other specific plant-derived bioactive molecules such as flavonoids and stilbenes that effectively target mechanisms involved in the initiation and development of prostate malignancies [83]. Moreover, the involvement of inflammation in the development of prostate cancer emphasises the potential of nonsteroidal anti-inflammatory drugs (NSAIDs) to decrease the risk. Actually, aspirin is a NSAID primarily used in cardiovascular diseases but its long-term use has been associated with reduced cancer risk [84]. Aspirin exerts chemopreventive activity against prostate cancer [85] and the use of aspirin has been observed to be inversely associated with prostate cancer mortality and case-fatality [86]. Moreover, inflammation is closely related to the action of cyclooxygenase (COX) that catalyses messenger molecules within the inflammatory pathway; COX-2 is expressed in several tissues during inflammation while over-expressed COX-2 is reported in human prostate cancer tissues when compared with benign tissues [87]. Importantly, men using NSAIDs, particularly anti-COX-2 activity, are associated with decreased risk of prostate cancer with the protective effects more pronounced in aggressive prostate cancer and in individuals with a personal history of prostatitis [87]. However, Zhou et al. recently observed no association between aspirin or non-aspirin NSAIDs and prostate cancer survival but described longer overall survival in the case of aspirin administration before and after the diagnosis of prostate cancer [88]. In addition, the enzymes and receptors of the lipoxygenase (LOX) pathway of arachidonic acid metabolism also play a notable role in prostate carcinogenesis affecting mechanisms such as cell proliferation, differentiation and apoptosis via multiple signalling pathways [89]. In this regard, a positive correlation between LOX inhibition and cytotoxicity of prostate cancer cells was found after the treatment with synthetic [90] or plant-derived [91] compounds. In summary, inflammation plays an essential role in the development and progression of prostate cancer [68,69] while natural substances or NSAIDs function as potent anti-inflammatory agents decreasing the overall severity of prostate cancer [78,79,84,85].

### 3.4. Hypoxia–Ischemia, Increased Endothelin-1 and the Protective Role of Phytochemicals and Nitric Oxide

Hypoxia is closely related to the development and aggressiveness of cancer including prostate cancer. Hypoxia-inducible factors (HIF) are master transcription factors that modulate genes responsive to hypoxia [92] that are critical for the adaptation and survival of cancer cells under hypoxic conditions [93] as well as for tumour invasiveness, chemo or radio resistance, angiogenesis and metastasis. Additionally, hypoxia enhances the phenotypes of cancer stem cells and EMT that are also implicated in therapy resistance [92,94]. Moreover, HIF-1α over-expression is reported in prostate carcinogenesis and metastasis. Indeed, the activity of Notch signalling is associated with the expression of HIF-1α and vascular endothelial growth factor (VEGF), the two markers of tumour hypoxia [95]. Furthermore, small regulatory peptides such as endothelin-1 exert mitogenic effects on prostate cancer progression through binding to the endothelin A receptor that results in the modulation of various kinases involved in cellular signalling [96]. Elevated levels of plasma concentrations of endothelin-1 are observed in men with metastatic prostate cancer [96]. Moreover, endothelin-1 functions also as an angiogenic factor; the density of micro-vessels and levels of endothelial growth factors are associated with endothelin-1 with further amplification under hypoxic conditions. Hypoxia signalling is potentiated by endothelin-1 through the regulation of HIF-1α; actually, there is a reciprocal relationship described by endothelin-1 stabilising HIF-1α that results in the activation of angiogenic genes regulated by HIF-1α including the transcription of endothelin-1 itself mediated by HIF-1α [97].

Nevertheless, naturally occurring phytochemicals exert a potent capacity to inhibit hypoxia-activated pathways associated with prostate cancer progression as demonstrated by bioflavonoid apigenin [98], silibinin [99] or quercetin in vitro and/or in vivo [100]. In addition, nitric oxide is a ubiquitous signalling molecule that plays an essential role in the human body. However, nitric oxide shows several effects on cancer to be kept in mind: low levels of nitric oxide are suggested to promote cancers, while high levels of nitric oxide exert protective effects against cancers [18]. Actually, high levels of nitric oxide produced by resident inflammatory macrophages can be damaging to cancer cells due to the conversion to pro-oxidants such as peroxynitrite and nitrogen dioxide [101]. As discussed above, tumour hypoxia leads to the adaptive phenotype of cancer cells that is mediated by various mechanisms; however, decreased nitric oxide-dependent signalling is importantly involved in the progression of malignant phenotypes. Invasiveness, metastatic abilities, evasion of immune cell recognition and therapy resistance induced by hypoxia (representing the adaptation of cancer cells in hypoxic condition) are inhibited by molecules that activate nitric oxide signalling. Indeed, inhibition of nitric oxide signalling leads to a phenotype similar to the phenotype induced by tumour hypoxia [102]. However, increased levels of nitric oxide result in the inhibition of the growth of prostate tumours in a cell non-autonomous manner in vivo [103]. Moreover, a phase II study of nitric oxide donors for men with increasing prostate-specific antigen levels after surgery or radiotherapy for prostate cancer demonstrated that nitric oxide attenuates hypoxia-induced progression of prostate cancer [102].

In conclusion, although hypoxia is closely associated with prostate cancer development and progression [92,93,94,97], naturally occurring phytochemicals possess the capacity to inhibit hypoxia-activated pathways required for carcinogenesis; also, nitric oxide functions as a potent inhibitor of prostate cancer [98,99,100,102,103].

### 3.5. Microbiome Profiling and a Protective Role of Probiotics in PCa Prevention

Increasing evidence links microbiota with human health and diseases, including cancer. Human microbiota is described as microorganisms, such as bacteria, archaea, protozoa or fungi physiologically living in the epithelial barrier of the body while the microbes with their genetic information represent the microbiome [104]. Under normal conditions, the microbiome encourages homeostasis and contributes to the immune responses [105] and also helps to maintain prostate health [105]. Alterations of microbiota associated with various stressors (age, diet, medications, smoking, exercise, diseases, environmental factors) termed as dysbiosis contribute to several pathologies including cancer [106]. In dysbiosis, the microbiome in multiple sites such as gastrointestinal or the urinary tract is suggested to be crucial for prostate cancer [104] through affecting immune responses and inflammation [106]. The microbiome also affects systemic hormone levels and is therefore important in the pathogenesis of prostate cancer that is affected by oestrogen and androgen levels [105]. The prostate is exposed to various inflammatory stimuli derived from bacteria of the urinary microbiome [104]. Moreover, profiling of the urinary microbiome revealed the prevalence of pro-inflammatory bacteria and uropathogens in the urinary tract of men with prostate cancer [107]. In addition to the urinary microbiome, normal and cancer prostate tissue is also associated with the presence of the microbiome [108]. Despite the role in inflammation, the microbiome can affect the prostate microenvironment while there is a pathophysiological association between the microbiome and prostate cancer [109,110]. Cavarretta et al. demonstrated differences in microbial populations among tumour/peri-tumour and non-tumour prostate specimens while *Staphylococcus* spp. were more represented in the tumour/peri-tumour tissues [109]. Therefore, the presence of a localised prostate-specific microbial profile can be regarded as a potentially novel marker of prostate cancer management [111]. In addition, studies also describe the relationship between gastrointestinal microbiota and prostate cancer risk [108]. Recently, differences in enrichments of *Bacteroides* and *Streptococcus* species in rectal swab samples have been identified in cancer while folate and arginine represented the most significantly altered pathways. Based on 10 aberrant metabolic pathways, these results allowed to form a novel microbiome-derived risk factor for prostate cancer [112]. Human microbiota is highly implicated also in the effectiveness of cancer treatment. For example, the profiling of faecal microbiota reveals a difference in alpha diversity in gastrointestinal diversity of microbiota in men with versus those without prostate cancer and in men receiving oral androgen receptor axis-targeted therapies. The results suggest that hormonal therapies of prostate cancer also alter the gastrointestinal microbiota and thus affect the response to such therapies and potentially modulate the anti-tumour effects of other therapies including immunotherapy [113]. In conclusion, the evaluation of the microbiome is essential for the purpose of personalised data regarding the presence or absence of specific species that are implicated in the evaluation of prostate cancer risk [112].

Human gut microbiome interplay has a critical role in the processes of inflammation and oncogenesis. Comprehensive research data demonstrated that inflammation increases the cancer risk. However, the microbial community of the gut that can be significantly affected by dietary supplementation with prebiotics and probiotics can suppress pro-inflammatory cell signalling and thus reduce the incidence of cancer [114]. The correction of microbiota problems can be performed using beneficial probiotics [115]. Indeed, probiotics are known for tumour-preventive characteristics in animal models [116,117,118]. Importantly, probiotic whey dairy beverages have been demonstrated to induce extensive apoptosis in human prostate cancer cell lines in vitro, regardless of the probiotic strain. However, further animal and human studies are required for precise evaluation of the potential of probiotic whey beverages in the therapy of prostate cancer [119]. Additionally, the impact of dietary preferences and obesity on prostate health is highlighted by Frugé et al. who describe the overweight and obese cancer patients to be dysbiotic and manifesting unique microbiome profiles [120]. Therefore, current research highlights the essential role of human microbiota in prostate cancer while the individual profile of a microbiome could serve as a potent biomarker of the disease course [112] and efficient modulation of human microbiota could improve the overall management of prostate cancer [121].

**Figure 1:**
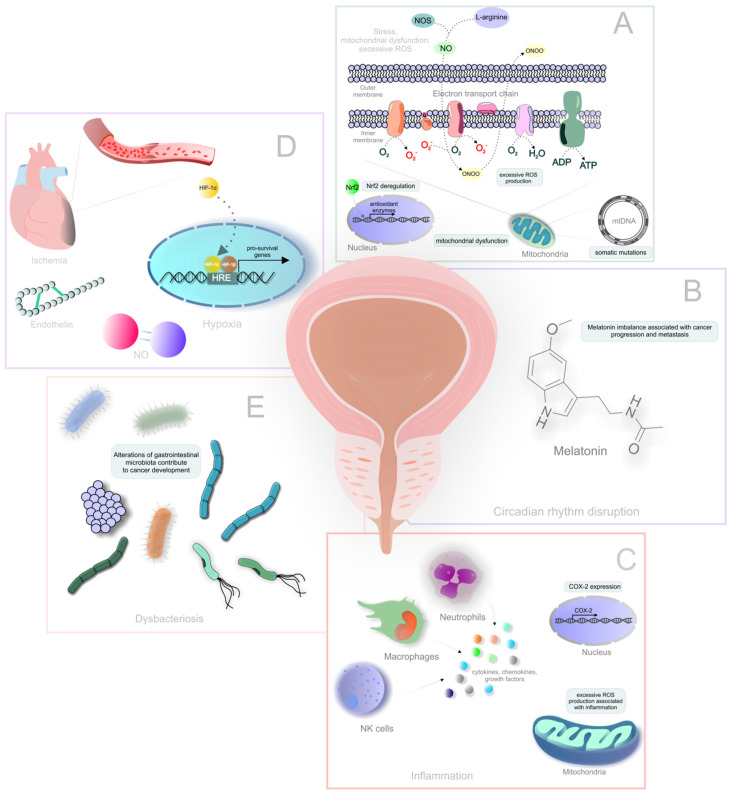
Systemic effects involved in PCa development and progression. Explanatory notes: (**A**) mitochondrial dysfunction, excessive ROS, oxidative stress; (**B**) circadian rhythm disruption; (**C**) inflammation; (**D**) hypoxia; (**E**) dysbacteriosis.

The systemic effects involved in PCa development and progression presented above are summarised in Figure 1.

## 4. Status Quo and Expert Recommendations in the Framework of 3P Medicine

As highlighted above, there are evident challenges currently observed in primary, secondary and tertiary PCa care. Here, we summarise recent achievements in the field followed by expert recommendations in the framework of 3P medicine [122].

### 4.1. PCa Risk Assessment: Application of Specialised Surveys Is of Clinical Relevance for the Disease Prevention and Management

**Figure 2:**
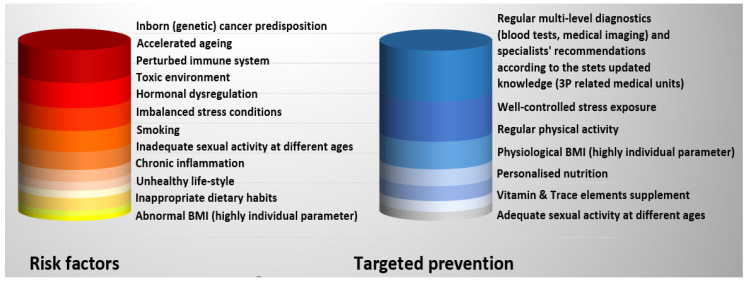
PCa-relevant risk assessment, individualised prediction and targeted prevention [3].

PCa relevant non-modifiable and modifiable risk factors are summarised in Figure 2 [3]. The corresponding survey is applicable to individualised patient profiling, patient stratification, evidence-based prediction and targeted prevention in the overall PCa management. According to evidence, toxic environment, stress overload, inadequate behavioural patterns, sub-optimal diet and metabolic impairments, amongst others, are modifiable risk factors that can be thoroughly analysed by surveys; corresponding data indicating potential risks should follow by the liquid biopsy-based non-invasive risk assessment that is of great value for targeted PCa prevention [3].

### 4.2. Liquid Biopsy Biomarker Panels for PCa Prediction, Prognosis and Patient Stratification: Status Quo and Outlook

PCa-relevant biomarker panels utilising liquid biopsy are exemplified in Table 1. Liquid biopsy samples make use of urine, whole blood, peripheral leucocytes, circulating tumour cells (CTC), blood serum, blood plasma and saliva. Disease- and stage-specific biomarker panels comprise CTCs, cell-free DNA, tumour-specific DNA, mRNA, miRNA, lncRNA, protein and metabolite patterns.

Serum prostate specific antigen (PSA) level is the gold standard biomarker for the clinical management of prostate cancer. However, PSA shows a low accuracy because of potential false-positive results, resulting in unnecessary invasive prostate biopsies, and up to 15% of undetected lesions. For instance, androgen receptor (AR) negative prostate cancer and neuroendocrine PCa do not express PSA. As a result, some peculiar lesions fail to be promptly identified with PSA serum blood tests. Moreover, the latest literature suggests that PCa aetiology and staging are strictly related to distinct metabolic, environmental and lifestyle factors. An outstanding example is the differential incidence and outcomes of PCa observed considering various ethnicities, behavioural patterns, metabolic particularities and comorbidities [3].

Considering the high accessibility and non-invasiveness of blood sampling, blood-based predictive and prognostic models are expanding in the clinically relevant PCa research. To this end, Hofmann et al. described a new multi-analyte assay, including CTCs enumeration and characterisation by mRNA-based in situ padlock probe technology, mRNA expression analysis of androgen receptor splice variant 7 (AR-V7), androgen receptor full length (AR-FL), and kallikrein related peptidase 3 (KLK3) transcripts from whole blood lysates by RT-qPCR, and detection of AR amplification by plasma-Seq [141].

CTCs enumeration per se had already demonstrated its great value as a predictive and prognostic biomarker; CTCs isolation can be easily achieved via positive or negative selection thanks to the expression of tumour-associated cell surface proteins, such as the epithelial cell adhesion molecule (EpCAM) or epidermal growth factor receptor (EGFR), and the lack of the leukocyte antigen cluster of differentiation 45 (CD45) [128]. Newly developed techniques for CTCs enumeration, such as FDA-cleared CellSearch^®^ system, the dual fluoro-EPISPOT assay and the in vivo CellCollector^®^ technology, have also been used efficiently to monitor residual or relapsing PCa after local therapy [138]. Despite the current techniques used on CTCs being mainly based on their physical or epithelial characteristics, evidence shows that further downstream investigations could be promising for clinical practice. Notably, Chen et al. took advantage of molecular biology techniques on CTCs to determine the metabolic marker (PGK1/G6PD) as an effective indicator of existing metastases [139]. A significant diagnostic value of high-risk PCa is carried also by the RT-qPCR panel established by Arkou et al., specifically investigating the expression of 14 genes (*KRT19*, *EpCAM*, *CDH1*, *HMBS*, *PSCA*, *ALDH1A1*, *PROM1*, *HPRT1*, *TWIST1*, *VIM*, *CDH2*, *B2M*, *PLS3* and *PSA*) [137]. On the other hand, the prognostic validity of CTC biomarkers is certainly the most disputed and several proteins (e.g., AR, AR-v7, vimentin, Ki67, albumin, lactate dehydrogenase, PSA, haemoglobin and alkaline phosphatase) have already been investigated to monitor the treatment response of metastatic castration-resistant prostate cancer (mCRPC) [128]. Notably, AR and AR-v7 turned out to be of high interest for the improvement of mCRPC prognosis. In fact, multiple studies relate their expressional level in CTCs with the differential response to second-generation AR-targeting agents and adequately encourage the rearrangement of the patients’ therapy towards a taxane treatment [124,128,134]. AR-v7, in particular, was detected also in exosomes and directly from blood. In both cases, it was associated with a significantly shorter overall survival (OS) [128].

There are very few examples amongst a wide range of studies performed using liquid biopsies on tumour cells products, which include exosomes as well as cell tumour DNA (ctDNA), cell free DNA (cfDNA), long non-coding RNA (lncRNA), extracellular vesicles, metabolites and miRNAs [124,129,143]. Interestingly, in blood isolated ctDNA were mostly assessed hyper-methylation markers (*T6GALNAC3*, *CCDC181*, *HAPLN3*) and the methylation level of several promoters (*APC*, *FOXA1*, *GSTP1*, *HOXD3*, *RARβ2*, *RASSF1A*, *SEPT9*, *SOX17 ST6GALNAC3* and *ZNF660*) for the early detection of PCa [124,125]. Some methylation panels were constructed for the investigation of ctDNA in urine samples as well [124,135,136]. Similar analyses were performed on plasma cfDNA, with the conclusive development of a minimally invasive test based on the detection of promoter methylation levels of *APCme*, *FOXA1me*, *GSTP1me*, *HOXD3me*, *RARβ2me*, *RASSF1Ame*, *SEPT9me* and *SOX17me* [133].

As such, cfDNA or RNA fragments released after the lysis of apoptotic or necrotic tumour cells can be used as the main targets for liquid biopsies in prostate cancer with specific biomarkers. Once the DNA is isolated, several genes and mutations can be detected. Moreover, promoter methylation levels of some genes can be also applied using multiplex quantitative methylation-specific PCR [133]. Because of the anatomy of the prostate, the above-mentioned markers can be also detected in seminal fluid or urine. For example, Ponti et al. [123] showed that cfDNA was significantly higher in PCa patients compared to benign prostate hyperplasia (BPH) patients and healthy controls. Additionally, seminal cfDNA fragments longer than 1000 base-pairs were more common in patients with PCa compared to those with BPH and controls. Since a property of prostate tumour cells is specific metabolic reprogramming, metabolic products can be utilised to characterise the functional activity of prostate CTCs. For example, eight metastasis-related metabolic genes were identified, including *HK2*, *PDP2*, *G6PD*, *PGK1*, *PHKA1*, *PYGL*, *PDK1* and *PKM2*. In the prostate, *PGK1* and *G6PD* were determined as optimal glucose metabolic markers for CTCs [139]. Further, a recent study published by Hashimoto K. and colleagues [144] provides strong evidence that disease-specific patterns detected in biopsies are generally useful for diagnosing bone and soft tissue metastases, for identifying their primary site that allows for targeted treatments and may lead to improved individual outcomes [144].

Another highly promising field is metabolomics in PCa prediction, diagnosis, progression, prognosis, patient stratification, therapy planning and monitoring. Metabolomics is considered an interdisciplinary “omics” approach combining pattern identification and bioinformatics with epidemiology, analytical biochemistry and disease biology [145]. PCa as a multi-factorial disease is a particularly attractive model for metabolite profiling linked with comprehensive health risk assessment [146], since

- there is strong evidence demonstrating that dysregulation of metabolism plays a crucial role in the development and progression of PCa;
- one of the most prominent risk factors is the metabolic syndrome: there is a synergic interplay of visceral obesity, insulin resistance, low HDL (high-density lipoprotein) cholesterol, high triglycerides, elevated C-reactive protein and low adiponectin levels, amongst others in the pathomechanisms of PCa;
- further, there are organ-specific particularities in the metabolism of the prostate to produce the components of prostatic fluid: PSA, spermine, myo-inositol and citrate; to this end, the levels of citrate in the prostate are orders of magnitude higher than anywhere else in the body;
- finally, neoplastic prostate cells lose the capacity to accumulate zinc which is thought to inhibit the ability to accumulate citrate; metabolomic alterations reflecting this unique phenomenon are hypothesised to result in a PCa-specific metabolome profile that might be instrumental for the disease modelling.

## Figures and Tables

**Table 1:** Liquid biopsy biomarker panels for PCa prediction, prognosis and patient stratification.

Type of Patient Stratification	Type of Biomarker	Characterisation	References
PCa versus benign adenomas	cfDNA	Cell free DNA in seminal fluid	[123]
		Clinical relevance: Diagnosis	
		Liquid biopsy: Seminal fluid	
	ctDNA	ct-DNA methylation panel: *ST6GALNAC3*, *CCDC181*, *HAPLN3*	[124,125]
		Clinical relevance: Diagnosis	
		Liquid biopsy: Serum	
	miRNA	1231 high-throughput miRNA-profiled serum samples	[126]
		The pairwise model was composed of five circulating miRNAs coupled to miR-5100 and miR-1290.	
		Clinical relevance: Diagnosis	
		Liquid biopsy: Serum	
	Proteins	Circulating inflammatory markers (Cytokines)-neutrophil/lymphocyte (NLR), neutrophil/monocyte (NMR) and platelet/lymphocyte (PLR)	[127]
		Clinical relevance: Diagnosis	
		Liquid biopsy: Blood	
	miRNA	mir-200-family (including miRNA-141-3p) and miR-375	[128]
		Clinical relevance: Prognosis	
		Liquid biopsy: Urine	
	cfDNA	Long non-coding RNAs- MALAT1-Metastasis Associated Lung Adenocarcinoma Transcript 1 (*MALAT1*)	[128]
		Clinical relevance: Diagnosis	
		Liquid biopsy: Blood plasma	
	miRNA	let-7c, miR-30c, miR-141, miR-375	[129]
		Clinical relevance: Diagnosis, treatment algorithms	
		Liquid biopsy: Blood plasma	
	miRNA	miR-572, miR-1290, miR-141 and miR-145 (in EV)	[129]
		Clinical relevance: Diagnosis	
		Liquid biopsy: Urine	
	miRNA	miR-21, miR-141, miR-214, miR-375 and let-7c (in EV)	[129]
		Clinical relevance: Diagnosis	
		Liquid biopsy: Urine	
	Proteins	ERG + PCA3 transcripts (ExoDx Prostate Intelliscore) (in EV)	[129]
		Clinical relevance: Diagnosis	
		Liquid biopsy: Urine	
	Proteins	Sensitivity and Specificity Analysis for Salivary PSA, B2M, CK-BB, MT, Zinc, Creatinine and Urea	[130]
		Clinical relevance: Diagnosis	
		Liquid biopsy: Saliva, blood serum	
	Proteins	Inflammatory biomarkers: Interleukin-6 Single Nucleotide Polymorphism	[131]
		Clinical relevance: Prediction, diagnosis	
		Liquid biopsy: Blood	
	cfDNA	ALU sequence quantification and integrity	[129]
		Clinical relevance: Prediction, prognosis	
		Liquid biopsy: Blood plasma	
	Metabolites	Acetoacetate, cystine, glutamate, lysine, tyrosine, lipids	[132]
		Clinical relevance: Prediction, diagnosis	
		Liquid biopsy: Blood serum	
	Metabolites	Metabolites-based disease modelling: dihyroxybutanoic acid and xylonic acid (upregulated), pyrimidine, xylopyranose and ribofuranoside (downregulated)	[132]
		Clinical relevance: Diagnosis	
		Liquid biopsy: Urine	
PCa with versus PCa without metastatic potential	cfDNA	DNA methylation	[133]
		Promotor methylation levels of *APCme*, *FOXA1me*, *GSTP1me*, *HOXD3me*, *RARβ2me*, *RASSF1Ame*, *SEPT9me* and *SOX17me*	
		Clinical relevance: Prediction, prognosis	
		Liquid biopsy: Blood plasma	
	CTC enumeration/molecular biological characterisation	AR-signalling-dependent cancer cells	[134]
		Clinical relevance: Treatment algorithms	
		Liquid biopsy: Blood	
	ctDNA	DNA methylation panel (epigenetic regulation)	[135]
		Gene panel—*GSTP1*, *SFRP2*, *IGFBP3*, *IGFBP7*, *APC*, *PTGS2*	
		Clinical relevance: Prognosis	
		Liquid biopsy: Urine	
	ctDNA	Methylation Gene panel—*ADCY4*, *AOX1*, *APC*, *CXCL14*, *EPHX3*, *GFRA2*, *GSTP1*, *HEMK1*, *HOXA7*, *HOXB5*, *HOXD3a*, *HOXD3b*, *HOXD9* *HOXD10*, *KIFC2*, *MOXD1*, *NEUROG3*, *NODAL*, *RASSF5*, *NSD1*	[124,136]
		Clinical relevance: Diagnosis	
		Liquid biopsy: Urine	
	CTC enumeration/molecular biological characterisation	Gene panel-(*KRT19*, *EpCAM*, *CDH1*, *HMBS*, *PSCA*, *ALDH1A1*, *PROM1*, *HPRT1*, *TWIST1*, *VIM*, *CDH2*, *B2M*, *PLS3* and *PSA*)	[124,137]
		Clinical relevance: Diagnosis, prognosis	
		Liquid biopsy: Blood	
	CTC enumeration/molecular biological characterisation	Circulating tumour cells (CTC) using the CellSearch system, dual fluoro-EPISPOT assay and CellCollector	[124,138]
		Clinical relevance: Prediction, prognosis	
		Liquid biopsy: Blood	
	CTC enumeration/molecular biological characterisation	Metabolic characterisation of CTCs in the peripheral blood of PCa patients *PGK1* and *G6PD* (GM markers)	[124,139]
		Clinical relevance: Diagnosis, prognosis	
		Liquid biopsy: Blood	
	Proteins	African American men (AAM) with PCa compared to healthy AAM; Disease specific protein patterns: Isoform 2 of Coiled-coil and C2 domain-containing protein 1A, Keratin, type I cytoskeletal 10, UPF0728 protein C10orf53, DnaJ homolog subfamily C member 13, Prothrombin, Apolipoprotein (a), Coiled-coil domain-containing protein 172.	[140]
		Clinical relevance: Diagnosis, prognosis	
		Liquid biopsy: Blood serum	
	CTC enumeration/molecular biological characterisation	CTC enumeration and characterisation-*AR-V7*, *AR-FL*, *KLK3* mRNA expression *AR-V7*, *AR-FL*, *KLK3* mRNA expression *AR* amplification in ct-DNA	[141]
		Clinical relevance: Diagnosis, prognosis	
		Liquid biopsy: CTCs, RNA from whole blood lysates and plasma DNA	
	CTC enumeration/molecular biological characterisation	(*KLK3*, *FOLH1*, *NPY*)-tumour derived biomarkers	[128]
		Clinical relevance: Prognosis	
		Liquid biopsy: Blood	
	cfDNA	*KLK3* and *TMPRSS2-ERG*	[128]
		Clinical relevance: Prognosis	
		Liquid biopsy: Urine and blood	
	cfDNA	*SCHLAP1* SWI/SNF COMPLEX Antagonist Associated with Prostate Cancer 1 (*SCHLAP1*) lncRNA	[128]
		Clinical relevance: Diagnosis	
		Liquid biopsy: Urine	
	cfDNA	*MALAT1*	[128]
		Metastasis Associated Lung Adenocarcinoma Transcript 1 (*MALAT1*)	
		Clinical relevance: Prediction, prognosis	
		Liquid biopsy: Blood plasma	
	CTC enumeration/molecular biological characterisation	Androgen receptor variant 7 protein (*AR-V7*)	[129]
		Clinical relevance: Prognosis	
		Liquid biopsy: Blood	
	CTC enumeration/molecular biological characterisation	mRNA of *PSA*, *PSMA* and *EGFR* in CTCs	[129]
		Clinical relevance: Treatment algorithms, prognosis	
		Liquid biopsy: Blood	
	CTC enumeration/molecular biological characterisation	Nuclear localisation of *AR-V7* in CTCs	[129]
		Clinical relevance: Treatment algorithms, prognosis	
		Liquid biopsy: Blood	
	CTC enumeration/molecular biological characterisation	Albumin, LDH, PSA, haemoglobin and ALK (ALPHA) in serum and CTC enumeration	[129]
		Clinical relevance: Prediction, prognosis	
		Liquid biopsy: Blood	
	CTC enumeration/molecular biological characterisation	CTC enumeration, stem cell-related genes (*ABCG2*, *PROM1* and *PSCA*) and EMT-related genes (*TWIST1* and vimentin) in PBMCs	[129]
		Clinical relevance: Prediction, prognosis	
		Liquid biopsy: Blood	
	CTC enumeration/molecular biological characterisation	CD117/c-kit, CD133/prominin-1, CD34, CD184/CXCR4 and EpCAM/CD326 in lymphocytes	[129]
		Clinical relevance: Treatment algorithms, prognosis	
		Liquid biopsy: Blood	
	CTC enumeration/molecular biological characterisation	CTC enumeration and *AR-V7* mRNA in CTCs	[129]
		Clinical relevance: Treatment algorithms, prognosis	
		Liquid biopsy: Blood	
	CTC enumeration/molecular biological characterisation	CTC enumeration and Ki67 and vimentin in CTCs	[129]
		Clinical relevance: Treatment algorithms, prognosis	
		Liquid biopsy: Blood	
	CTC enumeration/molecular biological characterisation	Telomerase activity in CTCs and CTC enumeration	[129]
		Clinical relevance: Diagnosis, treatment algorithms,	
		prognosis	
		Liquid biopsy: Blood	
	CTC enumeration/molecular biological characterisation	mRNA of *KLK3*, *KLK2*, *HOXB13*, *GRHL2* and *FOXA1* in whole blood and CTC enumeration	[129]
		Clinical relevance: Treatment algorithms	
		Liquid biopsy: Blood	
	CTC enumeration/molecular biological characterisation	mRNA of antioxidant genes (*GPX1* and *SOD2*) and prostate genes (*AR*, *cyclin B* and *bFGF*) in CTCs	[129]
		Clinical relevance: Prediction, prognosis	
		Liquid biopsy: Blood	
	CTC enumeration/molecular biological characterisation	mRNA of anti-oxidant genes (*GPX1*, *SOD2* and *TXNRD1*), epithelial gene (*CK20*) and organ-specific genes (*AR*, *PSA*, *PSMA*) in CTCs	[129]
		Clinical relevance: Prediction, prognosis	
		Liquid biopsy: Blood	
	cfDNA	Copy number of cancer-related genes, *AR* and CTC numeration	[129]
		Clinical relevance: Prediction, prognosis	
		Liquid biopsy: Blood plasma	
	cfDNA	cBMP6 mRNA, cf-DNA, apoptotic nucleosomes and H3K27me3	[129]
		Clinical relevance: Diagnosis	
		Liquid biopsy: Blood plasma	
	cfDNA	cfDNA quantification	[129]
		Clinical relevance: Prediction, prognosis	
		Liquid biopsy: Blood plasma	
	cfDNA	cfDNA quantification	[129]
		Clinical relevance: Prediction, prognosis	
		Liquid biopsy: Blood serum	
	cfDNA	AR copy number and 19 cancer associated genes	[129]
		Clinical relevance: Prognosis	
		Liquid biopsy: Blood plasma	
	Proteins	Prostate-specific transcripts such as KLK3, PCA3 and ERG; kidney- and bladder-specific transcripts in EVs	[129]
		Clinical relevance: Diagnosis	
		Liquid biopsy: Urine	
	Proteins	ADSV-TGM4 and CD63-GLPK5-SPHM-PSA-PAPP	[129]
		Clinical relevance: Diagnosis, prognosis	
		Liquid biopsy: Urine	
	CTC enumeration/molecular biological characterisation	PSMA in prostate microparticles and CTC enumeration	[129]
		Clinical relevance: Prediction, prognosis	
		Liquid biopsy: Blood plasma	
	miRNA	RNA copy numbers of ERG and PCA3 (EXO106 score)	[129]
		Clinical relevance: Diagnosis	
		Liquid biopsy: Urine	
	miRNA	Serum miR-141 and miR-37; and urine miR-107 and miR-574-3p	[129]
		Clinical relevance: Diagnosis, prognosis	
		Liquid biopsy: Serum, plasma and urine	
	Proteins	Interleukin-6 Single Nucleotide Polymorphism	[129]
		Clinical relevance: Prediction	
		Liquid biopsy: Blood	
	miRNA	lncRNA-p21	[129]
		Clinical relevance: Diagnosis	
		Liquid biopsy: Urine	
	Metabolites	sarcosine, choline, phosphocholine	[142]
		Liquid biopsy: Diagnosis	
		Liquid biopsy: Blood, urine	
	Metabolites	Biomarker profile: Lysophosphatidylcholines (LPC) with saturated fatty acid chains, serotonin, monoamine, aspartic acid (Asp) and ornithine	[132]
		Clinical relevance: Diagnosis, prognosis	
		Liquid biopsy: Blood serum	
	Metabolites	Sarcosine (sediment)	[132]
		Sarcosine (supernatant)	
		Clinical relevance: Diagnosis, prognosis	
		Liquid biopsy: Urine	
	Metabolites	Acylcarnitine and arachidonoyl amine	[132]
		Clinical relevance: Diagnosis	
		Liquid biopsy: Blood plasma	
	Metabolites	Ala, Ile, Orn, Lys (downregulated), Gln, Val, Trp and Arg (upregulated)	[132]
		Clinical relevance: Treatment monitoring, prognosis	
		Liquid biopsy: Blood plasma	
	Metabolites	Metabolites-based disease modelling: Pyrimidine, Creatinine, Purine, Glucopyranoside, Xylopyranose and Ribofuranoside (downregulated), Propenoic acid, Dihyroxybutanoic acid and Xylonic acid	[132]
		Clinical relevance: Diagnosis, prognosis	
		Liquid biopsy: Urine	
	Metabolites	Ureido isobutyric acid, indolylacryloyglycine, acetylvanilalinine 2-oxoglutarate	[132]
		Clinical relevance: Prognosis	
		Liquid biopsy: Urine	

Abbreviations: Beta-2 microglobulin (B2M); creatine kinase BB (CK-BB); extracellular vesicles (EV); glucose metabolism (GM); melatonin (MT); peripheral blood mononuclear cell (PBMC).

## References

[1] Siegel R.L., Miller K.D., Jemal A. (2016). Cancer Statistics, 2016. CA Cancer J. Clin..

[2] Ferlay J., Colombet M., Soerjomataram I., Dyba T., Randi G., Bettio M., Gavin A., Visser O., Bray F. (2018). Cancer Incidence and Mortality Patterns in Europe: Estimates for 40 Countries and 25 Major Cancers in 2018. Eur. J. Cancer.

[3] Kucera R., Pecen L., Topolcan O., Dahal A.R., Costigliola V., Giordano F.A., Golubnitschaja O. (2020). Prostate Cancer Management: Long-Term Beliefs, Epidemic Developments in the Early Twenty-First Century and 3PM Dimensional Solutions. EPMA J..

[4] Bhaskaran K., dos-Santos-Silva I., Leon D.A., Douglas I.J., Smeeth L. (2018). Association of BMI with Overall and Cause-Specific Mortality: A Population-Based Cohort Study of 3·6 Million Adults in the UK. Lancet Diabetes Endocrinol..

[5] Thalgott M., Kron M., Brath J.M., Ankerst D.P., Thompson I.M., Gschwend J.E., Herkommer K. (2018). Men with Family History of Prostate Cancer Have a Higher Risk of Disease Recurrence after Radical Prostatectomy. World J. Urol..

[6] Wu I., Modlin C.S. (2012). Disparities in Prostate Cancer in African American Men: What Primary Care Physicians Can Do. Cleve Clin. J. Med..

[7] Sellami M., Gasmi M., Denham J., Hayes L.D., Stratton D., Padulo J., Bragazzi N. (2018). Effects of Acute and Chronic Exercise on Immunological Parameters in the Elderly Aged: Can Physical Activity Counteract the Effects of Aging?. Front. Immunol..

[8] Taverna G., Seveso M., Giusti G., Hurle R., Graziotti P., Stifter S., Chiriva-Internati M., Grizzi F. (2014). Senescent Remodeling of the Innate and Adaptive Immune System in the Elderly Men with Prostate Cancer. Curr. Gerontol. Geriatr. Res..

[9] Ihle C.L., Owens P. (2020). Integrating the Immune Microenvironment of Prostate Cancer Induced Bone Disease. Mol. Carcinog..

[10] Hassan S., Karpova Y., Baiz D., Yancey D., Pullikuth A., Flores A., Register T., Cline J.M., D’Agostino R., Danial N. (2013). Behavioral Stress Accelerates Prostate Cancer Development in Mice. J. Clin. Investig..

[11] Shukla S., Srivastava J.K., Shankar E., Kanwal R., Nawab A., Sharma H., Bhaskaran N., Ponsky L.E., Fu P., MacLennan G.T. (2020). Oxidative Stress and Antioxidant Status in High-Risk Prostate Cancer Subjects. Diagnostics.

[12] Straif K., Baan R., Grosse Y., Secretan B., El Ghissassi F., Bouvard V., Altieri A., Benbrahim-Tallaa L., Cogliano V., WHO International Agency for Research on Cancer Monograph Working Group (2007). Carcinogenicity of Shift-Work, Painting, and Fire-Fighting. Lancet Oncol..

[13] Gapstur S.M., Diver W.R., Stevens V.L., Carter B.D., Teras L.R., Jacobs E.J. (2014). Work Schedule, Sleep Duration, Insomnia, and Risk of Fatal Prostate Cancer. Am. J. Prev. Med..

[14] Shen D., Ju L., Zhou F., Yu M., Ma H., Zhang Y., Liu T., Xiao Y., Wang X., Qian K. (2021). The Inhibitory Effect of Melatonin on Human Prostate Cancer. Cell Commun. Signal..

[15] Hood S.P., Cosma G., Foulds G.A., Johnson C., Reeder S., McArdle S.E., Khan M.A., Pockley A.G. (2020). Identifying Prostate Cancer and Its Clinical Risk in Asymptomatic Men Using Machine Learning of High Dimensional Peripheral Blood Flow Cytometric Natural Killer Cell Subset Phenotyping Data. eLife.

[16] Da Silveira R.A., Hermes C.L., Almeida T.C., Bochi G.V., De Bona K.S., Moretto M.B., Moresco R.N. (2014). Ischemia-Modified Albumin and Inflammatory Biomarkers in Patients with Prostate Cancer. Clin. Lab..

[17] Torres Crigna A., Link B., Samec M., Giordano F.A., Kubatka P., Golubnitschaja O. (2021). Endothelin-1 Axes in the Framework of Predictive, Preventive and Personalised (3P) Medicine. EPMA J..

[18] Soni Y., Softness K., Arora H., Ramasamy R. (2020). The Yin Yang Role of Nitric Oxide in Prostate Cancer. Am. J. Men’s Health.

[19] Qian S., Golubnitschaja O., Zhan X. (2019). Chronic Inflammation: Key Player and Biomarker-Set to Predict and Prevent Cancer Development and Progression Based on Individualized Patient Profiles. EPMA J..

[20] Koklesova L., Mazurakova A., Samec M., Biringer K., Samuel S.M., Büsselberg D., Kubatka P., Golubnitschaja O. (2021). Homocysteine Metabolism as the Target for Predictive Medical Approach, Disease Prevention, Prognosis, and Treatments Tailored to the Person. EPMA J..

[21] Collin S.M., Metcalfe C., Refsum H., Lewis S.J., Zuccolo L., Smith G.D., Chen L., Harris R., Davis M., Marsden G. (2010). Circulating Folate, Vitamin B12, Homocysteine, Vitamin B12 Transport Proteins, and Risk of Prostate Cancer: A Case-Control Study, Systematic Review, and Meta-Analysis. Cancer Epidemiol. Biomark. Prev..

[22] Xu J., Chang W.-S., Tsai C.-W., Bau D.-T., Davis J.W., Thompson T.C., Logothetis C.J., Gu J. (2020). Mitochondrial DNA Copy Number in Peripheral Blood Leukocytes Is Associated with Biochemical Recurrence in Prostate Cancer Patients in African Americans. Carcinogenesis.

[23] Liskova A., Samec M., Koklesova L., Kudela E., Kubatka P., Golubnitschaja O. (2021). Mitochondriopathies as a Clue to Systemic Disorders—Analytical Tools and Mitigating Measures in Context of Predictive, Preventive, and Personalized (3P) Medicine. Int. J. Mol. Sci..

[24] Sabharwal S.S., Schumacker P.T. (2014). Mitochondrial ROS in Cancer: Initiators, Amplifiers or an Achilles’ Heel?. Nat. Rev. Cancer.

[25] Xiao J., Cohen P., Stern M.C., Odedina F., Carpten J., Reams R. (2018). Mitochondrial Biology and Prostate Cancer Ethnic Disparity. Carcinogenesis.

[26] Paschos A., Pandya R., Duivenvoorden W.C.M., Pinthus J.H. (2013). Oxidative Stress in Prostate Cancer: Changing Research Concepts towards a Novel Paradigm for Prevention and Therapeutics. Prostate Cancer Prostatic Dis..

[27] Abbasi A., Mostafavi-Pour Z., Amiri A., Keshavarzi F., Nejabat N., Ramezani F., Sardarian A., Zal F. (2020). Chemoprevention of Prostate Cancer Cells by Vitamin C plus Quercetin: Role of Nrf2 in Inducing Oxidative Stress. Nutr. Cancer.

[28] Mirzaei S., Mohammadi A.T., Gholami M.H., Hashemi F., Zarrabi A., Zabolian A., Hushmandi K., Makvandi P., Samec M., Liskova A. (2021). Nrf2 Signaling Pathway in Cisplatin Chemotherapy: Potential Involvement in Organ Protection and Chemoresistance. Pharm. Res..

[29] Tan B.L., Norhaizan M.E. (2021). Oxidative Stress, Diet and Prostate Cancer. World J. Men’s Health.

[30] Oh B., Figtree G., Costa D., Eade T., Hruby G., Lim S., Elfiky A., Martine N., Rosenthal D., Clarke S. (2016). Oxidative Stress in Prostate Cancer Patients: A Systematic Review of Case Control Studies. Prostate Int..

[31] Applegate C.C., Rowles J.L., Ranard K.M., Jeon S., Erdman J.W. (2018). Soy Consumption and the Risk of Prostate Cancer: An Updated Systematic Review and Meta-Analysis. Nutrients.

[32] Kubatka P., Uramova S., Kello M., Kajo K., Samec M., Jasek K., Vybohova D., Liskova A., Mojzis J., Adamkov M. (2019). Anticancer Activities of *Thymus Vulgaris* L. in Experimental Breast Carcinoma In Vivo and In Vitro. Int. J. Mol. Sci..

[33] Kubatka P., Kello M., Kajo K., Kruzliak P., Výbohová D., Mojžiš J., Adamkov M., Fialová S., Veizerová L., Zulli A. (2017). Oregano Demonstrates Distinct Tumour-Suppressive Effects in the Breast Carcinoma Model. Eur. J. Nutr..

[34] Kubatka P., Kello M., Kajo K., Samec M., Jasek K., Vybohova D., Uramova S., Liskova A., Sadlonova V., Koklesova L. (2020). Chemopreventive and Therapeutic Efficacy of *Cinnamomum zeylanicum* L. Bark in Experimental Breast Carcinoma: Mechanistic In Vivo and In Vitro Analyses. Molecules.

[35] Kapinova A., Kubatka P., Liskova A., Baranenko D., Kruzliak P., Matta M., Büsselberg D., Malicherova B., Zulli A., Kwon T.K. (2019). Controlling Metastatic Cancer: The Role of Phytochemicals in Cell Signaling. J. Cancer Res. Clin. Oncol..

[36] Koklesova L., Liskova A., Samec M., Qaradakhi T., Zulli A., Smejkal K., Kajo K., Jakubikova J., Behzadi P., Pec M. (2020). Genoprotective Activities of Plant Natural Substances in Cancer and Chemopreventive Strategies in the Context of 3P Medicine. EPMA J..

[37] Samec M., Liskova A., Kubatka P., Uramova S., Zubor P., Samuel S.M., Zulli A., Pec M., Bielik T., Biringer K. (2019). The Role of Dietary Phytochemicals in the Carcinogenesis via the Modulation of MiRNA Expression. J. Cancer Res. Clin. Oncol..

[38] Abotaleb M., Liskova A., Kubatka P., Büsselberg D. (2020). Therapeutic Potential of Plant Phenolic Acids in the Treatment of Cancer. Biomolecules.

[39] Zhai K., Siddiqui M., Abdellatif B., Liskova A., Kubatka P., Büsselberg D. (2021). Natural Compounds in Glioblastoma Therapy: Preclinical Insights, Mechanistic Pathways, and Outlook. Cancers.

[40] Zhai K., Brockmüller A., Kubatka P., Shakibaei M., Büsselberg D. (2020). Curcumin’s Beneficial Effects on Neuroblastoma: Mechanisms, Challenges, and Potential Solutions. Biomolecules.

[41] Liskova A., Samec M., Koklesova L., Brockmueller A., Zhai K., Abdellatif B., Siddiqui M., Biringer K., Kudela E., Pec M. (2021). Flavonoids as an Effective Sensitizer for Anti-Cancer Therapy: Insights into Multi-Faceted Mechanisms and Applicability towards Individualized Patient Profiles. EPMA J..

[42] Siddiqui M., Abdellatif B., Zhai K., Liskova A., Kubatka P., Büsselberg D. (2021). Flavonoids Alleviate Peripheral Neuropathy Induced by Anticancer Drugs. Cancers.

[43] Sharmila G., Athirai T., Kiruthiga B., Senthilkumar K., Elumalai P., Arunkumar R., Arunakaran J. (2014). Chemopreventive Effect of Quercetin in MNU and Testosterone Induced Prostate Cancer of Sprague-Dawley Rats. Nutr. Cancer.

[44] Rowles J.L., Ranard K.M., Smith J.W., An R., Erdman J.W. (2017). Increased Dietary and Circulating Lycopene Are Associated with Reduced Prostate Cancer Risk: A Systematic Review and Meta-Analysis. Prostate Cancer Prostatic Dis..

[45] Rowles J.L., Ranard K.M., Applegate C.C., Jeon S., An R., Erdman J.W. (2018). Processed and Raw Tomato Consumption and Risk of Prostate Cancer: A Systematic Review and Dose-Response Meta-Analysis. Prostate Cancer Prostatic Dis..

[46] Wang G., Zhang D., Yang S., Wang Y., Tang Z., Fu X. (2018). Co-Administration of Genistein with Doxorubicin-Loaded Polypeptide Nanoparticles Weakens the Metastasis of Malignant Prostate Cancer by Amplifying Oxidative Damage. Biomater. Sci..

[47] Khandrika L., Kumar B., Koul S., Maroni P., Koul H.K. (2009). Role of Oxidative Stress in Prostate Cancer. Cancer Lett..

[48] Wang C., Shu L., Zhang C., Li W., Wu R., Guo Y., Yang Y., Kong A.-N. (2018). Histone Methyltransferase Setd7 Regulates Nrf2 Signaling Pathway by Phenethyl Isothiocyanate and Ursolic Acid in Human Prostate Cancer Cells. Mol. Nutr. Food Res..

[49] Wendeu-Foyet M.G., Menegaux F. (2017). Circadian Disruption and Prostate Cancer Risk: An Updated Review of Epidemiological Evidences. Cancer Epidemiol. Biomark. Prev..

[50] Sulli G., Lam M.T.Y., Panda S. (2019). Interplay between Circadian Clock and Cancer: New Frontiers for Cancer Treatment. Trends Cancer.

[51] Papantoniou K., Castaño-Vinyals G., Espinosa A., Aragonés N., Pérez-Gómez B., Burgos J., Gómez-Acebo I., Llorca J., Peiró R., Jimenez-Moleón J.J. (2015). Night Shift Work, Chronotype and Prostate Cancer Risk in the MCC-Spain Case-Control Study. Int. J. Cancer.

[52] Markt S.C., Valdimarsdottir U.A., Shui I.M., Sigurdardottir L.G., Rider J.R., Tamimi R.M., Batista J.L., Haneuse S., Flynn-Evans E., Lockley S.W. (2015). Circadian Clock Genes and Risk of Fatal Prostate Cancer. Cancer Causes Control.

[53] Ashrafizadeh M., Najafi M., Kavyiani N., Mohammadinejad R., Farkhondeh T., Samarghandian S. (2021). Anti-Inflammatory Activity of Melatonin: A Focus on the Role of NLRP3 Inflammasome. Inflammation.

[54] Maestroni G.J.M., Bartsch C., Bartsch H., Blask D.E., Cardinali D.P., Hrushesky W.J.M., Mecke D. (2001). Melatonin and the immune system therapeutic potential in cancer, viral diseases, and immunodeficiency states. The Pineal Gland and Cancer: Neuroimmunoendocrine Mechanisms in Malignancy.

[55] Gatti G., Lucini V., Dugnani S., Calastretti A., Spadoni G., Bedini A., Rivara S., Mor M., Canti G., Scaglione F. (2017). Antiproliferative and Pro-Apoptotic Activity of Melatonin Analogues on Melanoma and Breast Cancer Cells. Oncotarget.

[56] Cheng J., Yang H.-L., Gu C.-J., Liu Y.-K., Shao J., Zhu R., He Y.-Y., Zhu X.-Y., Li M.-Q. (2019). Melatonin Restricts the Viability and Angiogenesis of Vascular Endothelial Cells by Suppressing HIF-1α/ROS/VEGF. Int. J. Mol. Med..

[57] Kubatka P., Zubor P., Busselberg D., Kwon T.K., Adamek M., Petrovic D., Opatrilova R., Gazdikova K., Caprnda M., Rodrigo L. (2018). Melatonin and Breast Cancer: Evidences from Preclinical and Human Studies. Crit. Rev. Oncol. Hematol..

[58] Bojková B., Kubatka P., Qaradakhi T., Zulli A., Kajo K. (2018). Melatonin May Increase Anticancer Potential of Pleiotropic Drugs. Int. J. Mol. Sci..

[59] Orendas P., Kubatka P., Kajo K., Stollarova N., Kassayova M., Bojkova B., Pec M., Nosal V., Kiskova T., Zihlavnikova K. (2012). Melatonin Enhanced Bexarotene Efficacy in Experimental Mammary Carcinogenesis. Neoplasma.

[60] Kubatka P., Bojková B., Kassayová M., Orendáš P., Kajo K., Výbohová D., Kružliak P., Adamicová K., Péč M., Stollárová N. (2014). Combination of Pitavastatin and Melatonin Shows Partial Antineoplastic Effects in a Rat Breast Carcinoma Model. Acta Histochem..

[61] Orendáš P., Kubatka P., Bojková B., Kassayová M., Kajo K., Výbohová D., Kružliak P., Péč M., Adamkov M., Kapinová A. (2014). Melatonin Potentiates the Anti-Tumour Effect of Pravastatin in Rat Mammary Gland Carcinoma Model. Int. J. Exp. Pathol..

[62] Bojková B., Kajo K., Kubatka P., Solár P., Péč M., Adamkov M. (2019). Metformin and Melatonin Improve Histopathological Outcome of NMU-Induced Mammary Tumors in Rats. Pathol. Res. Pract..

[63] Tai S.-Y., Huang S.-P., Bao B.-Y., Wu M.-T. (2016). Urinary Melatonin-Sulfate/Cortisol Ratio and the Presence of Prostate Cancer: A Case-Control Study. Sci. Rep..

[64] Sigurdardottir L.G., Markt S.C., Rider J.R., Haneuse S., Fall K., Schernhammer E.S., Tamimi R.M., Flynn-Evans E., Batista J.L., Launer L. (2015). Urinary Melatonin Levels, Sleep Disruption, and Risk of Prostate Cancer in Elderly Men. Eur. Urol..

[65] Wang S.-W., Tai H.-C., Tang C.-H., Lin L.-W., Lin T.-H., Chang A.-C., Chen P.-C., Chen Y.-H., Wang P.-C., Lai Y.-W. (2021). Melatonin Impedes Prostate Cancer Metastasis by Suppressing MMP-13 Expression. J. Cell Physiol..

[66] Zharinov G.M., Bogomolov O.A., Chepurnaya I.V., Neklasova N.Y., Anisimov V.N. (2020). Melatonin Increases Overall Survival of Prostate Cancer Patients with Poor Prognosis after Combined Hormone Radiation Treatment. Oncotarget.

[67] Tewari A.K., Stockert J.A., Yadav S.S., Yadav K.K., Khan I. (2018). Inflammation and Prostate Cancer. Adv. Exp. Med. Biol..

[68] Gurel B., Lucia M.S., Thompson I.M., Goodman P.J., Tangen C.M., Kristal A.R., Parnes H.L., Hoque A., Lippman S.M., Sutcliffe S. (2014). Chronic Inflammation in Benign Prostate Tissue Is Associated with High-Grade Prostate Cancer in the Placebo Arm of the Prostate Cancer Prevention Trial. Cancer Epidemiol. Biomark. Prev..

[69] Stark T., Livas L., Kyprianou N. (2015). Inflammation in Prostate Cancer Progression and Therapeutic Targeting. Transl. Urol..

[70] Taverna G., Pedretti E., Di Caro G., Borroni E.M., Marchesi F., Grizzi F. (2015). Inflammation and Prostate Cancer: Friends or Foe?. Inflamm. Res..

[71] Rani A., Dasgupta P., Murphy J.J. (2019). Prostate Cancer: The Role of Inflammation and Chemokines. Am. J. Pathol..

[72] Abotaleb M., Samuel S.M., Varghese E., Varghese S., Kubatka P., Liskova A., Büsselberg D. (2018). Flavonoids in Cancer and Apoptosis. Cancers.

[73] Liskova A., Kubatka P., Samec M., Zubor P., Mlyncek M., Bielik T., Samuel S.M., Zulli A., Kwon T.K., Büsselberg D. (2019). Dietary Phytochemicals Targeting Cancer Stem Cells. Molecules.

[74] Liskova A., Koklesova L., Samec M., Smejkal K., Samuel S.M., Varghese E., Abotaleb M., Biringer K., Kudela E., Danko J. (2020). Flavonoids in Cancer Metastasis. Cancers.

[75] Liskova A., Koklesova L., Samec M., Varghese E., Abotaleb M., Samuel S.M., Smejkal K., Biringer K., Petras M., Blahutova D. (2020). Implications of Flavonoids as Potential Modulators of Cancer Neovascularity. J. Cancer Res. Clin. Oncol..

[76] Koklesova L., Liskova A., Samec M., Buhrmann C., Samuel S.M., Varghese E., Ashrafizadeh M., Najafi M., Shakibaei M., Büsselberg D. (2020). Carotenoids in Cancer Apoptosis-The Road from Bench to Bedside and Back. Cancers.

[77] Kapinova A., Stefanicka P., Kubatka P., Zubor P., Uramova S., Kello M., Mojzis J., Blahutova D., Qaradakhi T., Zulli A. (2017). Are Plant-Based Functional Foods Better Choice against Cancer than Single Phytochemicals? A Critical Review of Current Breast Cancer Research. Biomed. Pharmacother..

[78] Jiang L.-N., Liu Y.-B., Li B.-H. (2019). Lycopene Exerts Anti-Inflammatory Effect to Inhibit Prostate Cancer Progression. Asian J..

[79] Vogt T.M., Mayne S.T., Graubard B.I., Swanson C.A., Sowell A.L., Schoenberg J.B., Swanson G.M., Greenberg R.S., Hoover R.N., Hayes R.B. (2002). Serum Lycopene, Other Serum Carotenoids, and Risk of Prostate Cancer in US Blacks and Whites. Am. J. Epidemiol..

[80] Lu Q.-Y., Hung J.-C., Heber D., Go V.L.W., Reuter V.E., Cordon-Cardo C., Scher H.I., Marshall J.R., Zhang Z.-F. (2001). Inverse Associations between Plasma Lycopene and Other Carotenoids and Prostate Cancer. Cancer Epidemiol. Biomark. Prev..

[81] Kirsh V.A., Mayne S.T., Peters U., Chatterjee N., Leitzmann M.F., Dixon L.B., Urban D.A., Crawford E.D., Hayes R.B. (2006). A Prospective Study of Lycopene and Tomato Product Intake and Risk of Prostate Cancer. Cancer Epidemiol. Biomark. Prev..

[82] Peters U., Leitzmann M.F., Chatterjee N., Wang Y., Albanes D., Gelmann E.P., Friesen M.D., Riboli E., Hayes R.B. (2007). Serum Lycopene, Other Carotenoids, and Prostate Cancer Risk: A Nested Case-Control Study in the Prostate, Lung, Colorectal, and Ovarian Cancer Screening Trial. Cancer Epidemiol. Biomark. Prev..

[83] Oczkowski M., Dziendzikowska K., Pasternak-Winiarska A., Włodarek D., Gromadzka-Ostrowska J. (2021). Dietary Factors and Prostate Cancer Development, Progression, and Reduction. Nutrients.

[84] Khalaf N., Yuan C., Hamada T., Cao Y., Babic A., Morales-Oyarvide V., Kraft P., Ng K., Giovannucci E., Ogino S. (2018). Regular Use of Aspirin or Non-Aspirin Nonsteroidal Anti-Inflammatory Drugs Is Not Associated with Risk of Incident Pancreatic Cancer in Two Large Cohort Studies. Gastroenterology.

[85] Salinas C.A., Kwon E.M., FitzGerald L.M., Feng Z., Nelson P.S., Ostrander E.A., Peters U., Stanford J.L. (2010). Use of Aspirin and Other Nonsteroidal Antiinflammatory Medications in Relation to Prostate Cancer Risk. Am. J. Epidemiol..

[86] Hurwitz L.M., Joshu C.E., Barber J.R., Prizment A.E., Vitolins M.Z., Jones M.R., Folsom A.R., Han M., Platz E.A. (2019). Aspirin and Non-Aspirin NSAID Use and Prostate Cancer Incidence, Mortality, and Case-Fatality in the Atherosclerosis Risk in Communities Study. Cancer Epidemiol. Biomark. Prev..

[87] Doat S., Cénée S., Trétarre B., Rebillard X., Lamy P.-J., Bringer J.-P., Iborra F., Murez T., Sanchez M., Menegaux F. (2017). Nonsteroidal Anti-Inflammatory Drugs (NSAIDs) and Prostate Cancer Risk: Results from the EPICAP Study. Cancer Med..

[88] Zhou C.K., Daugherty S.E., Liao L.M., Freedman N.D., Abnet C.C., Pfeiffer R., Cook M.B. (2017). Do Aspirin and Other NSAIDs Confer a Survival Benefit in Men Diagnosed with Prostate Cancer? A Pooled Analysis of NIH-AARP and PLCO Cohorts. Cancer Prev. Res..

[89] Vishnupriya P., Aparna A., Vijaya Padma V. (2021). Lipoxygenase (LOX) Pathway: A Promising Target to Combat Cancer. Curr. Pharm. Des..

[90] Goftari S.N., Sadeghian H., Bahrami A.R., Maleki F., Matin M.M. (2019). Stylosin and Some of Its Synthetic Derivatives Induce Apoptosis in Prostate Cancer Cells as 15-Lipoxygenase Enzyme Inhibitors. Naunyn Schmiedebergs Arch. Pharm..

[91] Yarla N.S., Azad R., Basha M., Rajack A., Kaladhar D.S.V.G.K., Allam B.K., Pragada R.R., Singh K.N., Sunanda K.K., Pallu R. (2015). 5-Lipoxygenase and Cyclooxygenase Inhibitory Dammarane Triterpenoid 1 from Borassus Flabellifer Seed Coat Inhibits Tumor Necrosis Factor-α Secretion in LPSInduced THP-1 Human Monocytes and Induces Apoptosis in MIA PaCa-2 Pancreatic Cancer Cells. Anticancer Agents Med. Chem..

[92] Bao B., Ahmad A., Kong D., Ali S., Azmi A.S., Li Y., Banerjee S., Padhye S., Sarkar F.H. (2012). Hypoxia Induced Aggressiveness of Prostate Cancer Cells Is Linked with Deregulated Expression of VEGF, IL-6 and MiRNAs That Are Attenuated by CDF. PLoS ONE.

[93] Cohen M., Amir S., Golan M., Ben-Neriah Y., Mabjeesh N.J. (2019). β-TrCP Upregulates HIF-1 in Prostate Cancer Cells. Prostate.

[94] Ma Y., Liang D., Liu J., Axcrona K., Kvalheim G., Stokke T., Nesland J.M., Suo Z. (2011). Prostate Cancer Cell Lines under Hypoxia Exhibit Greater Stem-Like Properties. PLoS ONE.

[95] Marignol L., Rivera-Figueroa K., Lynch T., Hollywood D. (2013). Hypoxia, Notch Signalling, and Prostate Cancer. Nat. Rev. Urol..

[96] Whyteside A.R.W.R., Hinsley E.E.H.E., Lambert L.A.L.A., McDermott P.J.M.J., Turner A.J.T.J. (2010). ECE-1 Influences Prostate Cancer Cell Invasion via ET-1-Mediated FAK Phosphorylation and ET-1-Independent MechanismsThis Article Is One of a Selection of Papers Published in the Two-Part Special Issue Entitled 20 Years of Endothelin Research. Can. J. Physiol. Pharmacol..

[97] Wang R., Dashwood R.H. (2011). Endothelins and Their Receptors in Cancer: Identification of Therapeutic Targets. Pharm. Res..

[98] Mirzoeva S., Kim N.D., Chiu K., Franzen C.A., Bergan R.C., Pelling J.C. (2008). Inhibition of HIF-1 Alpha and VEGF Expression by the Chemopreventive Bioflavonoid Apigenin Is Accompanied by Akt Inhibition in Human Prostate Carcinoma PC3-M Cells. Mol. Carcinog..

[99] Deep G., Kumar R., Nambiar D.K., Jain A.K., Ramteke A.M., Serkova N.J., Agarwal C., Agarwal R. (2017). Silibinin Inhibits Hypoxia-Induced HIF-1α-Mediated Signaling, Angiogenesis and Lipogenesis in Prostate Cancer Cells: In Vitro Evidence and in Vivo Functional Imaging and Metabolomics. Mol. Carcinog..

[100] Lee D.-H., Lee Y.J. (2008). Quercetin Suppresses Hypoxia-Induced Accumulation of Hypoxia-Inducible Factor-1alpha (HIF-1alpha) through Inhibiting Protein Synthesis. J. Cell. Biochem..

[101] Fahey J.M., Girotti A.W. (2015). Accelerated Migration and Invasion Of Prostate Cancer Cells After a Photodynamic Therapy-Like Challenge: Role of Nitric OxidE. Nitric Oxide.

[102] Siemens D.R., Heaton J.P.W., Adams M.A., Kawakami J., Graham C.H. (2009). Phase II Study of Nitric Oxide Donor for Men with Increasing Prostate-Specific Antigen Level after Surgery or Radiotherapy for Prostate Cancer. Urology.

[103] Arora H., Panara K., Kuchakulla M., Kulandavelu S., Burnstein K.L., Schally A.V., Hare J.M., Ramasamy R. (2018). Alterations of Tumor Microenvironment by Nitric Oxide Impedes Castration-Resistant Prostate Cancer Growth. Proc. Natl. Acad. Sci. USA.

[104] Massari F., Mollica V., Di Nunno V., Gatto L., Santoni M., Scarpelli M., Cimadamore A., Lopez-Beltran A., Cheng L., Battelli N. (2019). The Human Microbiota and Prostate Cancer: Friend or Foe?. Cancers.

[105] Porter C.M., Shrestha E., Peiffer L.B., Sfanos K.S. (2018). The Microbiome in Prostate Inflammation and Prostate Cancer. Prostate Cancer Prostatic Dis..

[106] Wheeler K.M., Liss M.A. (2019). The Microbiome and Prostate Cancer Risk. Curr. Urol. Rep..

[107] Shrestha E., White J.R., Yu S.-H., Kulac I., Ertunc O., De Marzo A.M., Yegnasubramanian S., Mangold L.A., Partin A.W., Sfanos K.S. (2018). Profiling the Urinary Microbiome in Men with Positive versus Negative Biopsies for Prostate Cancer. J. Urol..

[108] Katongole P., Sande O.J., Joloba M., Reynolds S.J., Niyonzima N. (2020). The Human Microbiome and Its Link in Prostate Cancer Risk and Pathogenesis. Infect. Agent Cancer.

[109] Cavarretta I., Ferrarese R., Cazzaniga W., Saita D., Lucianò R., Ceresola E.R., Locatelli I., Visconti L., Lavorgna G., Briganti A. (2017). The Microbiome of the Prostate Tumor Microenvironment. Eur. Urol..

[110] Ma X., Chi C., Fan L., Dong B., Shao X., Xie S., Li M., Xue W. (2019). The Microbiome of Prostate Fluid Is Associated With Prostate Cancer. Front. Microbiol..

[111] Manzoor M.A.P., Rekha P.-D. (2017). Microbiome—The “Unforeseen Organ”. Nat. Rev. Urol..

[112] Liss M.A., White J.R., Goros M., Gelfond J., Leach R., Johnson-Pais T., Lai Z., Rourke E., Basler J., Ankerst D. (2018). Metabolic Biosynthesis Pathways Identified from Fecal Microbiome Associated with Prostate Cancer. Eur. Urol..

[113] Sfanos K.S., Markowski M.C., Peiffer L.B., Ernst S.E., White J.R., Pienta K.J., Antonarakis E.S., Ross A.E. (2018). Compositional Differences in Gastrointestinal Microbiota in Prostate Cancer Patients Treated with Androgen Axis-Targeted Therapies. Prostate Cancer Prostatic Dis..

[114] Jiang Z., Li L., Chen J., Wei G., Ji Y., Chen X., Liu J., Huo J. (2021). Human Gut-Microbiome Interplay: Analysis of Clinical Studies for the Emerging Roles of Diagnostic Microbiology in Inflammation, Oncogenesis and Cancer Management. Infect. Genet. Evol..

[115] Suvorov A. (2013). Gut Microbiota, Probiotics, and Human Health. Biosci. Microbiota Food Health.

[116] Sha S., Ni L., Stefil M., Dixon M., Mouraviev V. (2020). The Human Gastrointestinal Microbiota and Prostate Cancer Development and Treatment. Investig. Clin. Urol..

[117] Kassayová M., Bobrov N., Strojný L., Orendáš P., Demečková V., Jendželovský R., Kubatka P., Kisková T., Kružliak P., Adamkov M. (2016). Anticancer and Immunomodulatory Effects of Lactobacillus Plantarum LS/07, Inulin and Melatonin in NMU-Induced Rat Model of Breast Cancer. Anticancer Res..

[118] Kassayová M., Bobrov N., Strojný L., Kisková T., Mikeš J., Demečková V., Orendáš P., Bojková B., Péč M., Kubatka P. (2014). Preventive Effects of Probiotic Bacteria Lactobacillus Plantarum and Dietary Fiber in Chemically-Induced Mammary Carcinogenesis. Anticancer Res..

[119] Rosa L.S., Santos M.L., Abreu J.P., Balthazar C.F., Rocha R.S., Silva H.L.A., Esmerino E.A., Duarte M.C.K.H., Pimentel T.C., Freitas M.Q. (2020). Antiproliferative and Apoptotic Effects of Probiotic Whey Dairy Beverages in Human Prostate Cell Lines. Food Res. Int..

[120] Frugé A.D., Ptacek T., Tsuruta Y., Morrow C.D., Azrad M., Desmond R.A., Hunter G.R., Rais-Bahrami S., Demark-Wahnefried W. (2018). Dietary Changes Impact the Gut Microbe Composition in Overweight and Obese Men with Prostate Cancer Undergoing Radical Prostatectomy. J. Acad. Nutr. Diet.

[121] Mandair D., Rossi R.E., Pericleous M., Whyand T., Caplin M.E. (2014). Prostate Cancer and the Influence of Dietary Factors and Supplements: A Systematic Review. Nutr. Metab..

[122] Golubnitschaja O., Baban B., Boniolo G., Wang W., Bubnov R., Kapalla M., Krapfenbauer K., Mozaffari M.S., Costigliola V. (2016). Medicine in the Early Twenty-First Century: Paradigm and Anticipation—EPMA Position Paper 2016. EPMA J..

[123] Ponti G., Maccaferri M., Manfredini M., Micali S., Torricelli F., Milandri R., Del Prete C., Ciarrocchi A., Ruini C., Benassi L. (2019). Quick Assessment of Cell-Free DNA in Seminal Fluid and Fragment Size for Early Non-Invasive Prostate Cancer Diagnosis. Clin. Chim. Acta.

[124] Heidrich I., Ačkar L., Mossahebi Mohammadi P., Pantel K. (2021). Liquid Biopsies: Potential and Challenges. Int. J. Cancer.

[125] Haldrup C., Pedersen A.L., Øgaard N., Strand S.H., Høyer S., Borre M., Ørntoft T.F., Sørensen K.D. (2018). Biomarker Potential of ST6GALNAC3 and ZNF660 Promoter Hypermethylation in Prostate Cancer Tissue and Liquid Biopsies. Mol. Oncol..

[126] Liu H.-P., Lai H.-M., Guo Z. (2021). Prostate Cancer Early Diagnosis: Circulating MicroRNA Pairs Potentially beyond Single MicroRNAs upon 1231 Serum Samples. Brief. Bioinform..

[127] Espinoza A.R., Lavi J., Otaño N., Arenilla W., León R., Espinoza A., Salvador N., León A. (2019). Serum cellular inflammatory markers in the diagnosis of prostate cancer. Arch. Esp. Urol..

[128] Boerrigter E., Groen L.N., Van Erp N.P., Verhaegh G.W., Schalken J.A. (2020). Clinical Utility of Emerging Biomarkers in Prostate Cancer Liquid Biopsies. Expert Rev. Mol. Diagn..

[129] Campos-Fernández E., Barcelos L.S., de Souza A.G., Goulart L.R., Alonso-Goulart V. (2019). Research Landscape of Liquid Biopsies in Prostate Cancer. Am. J. Cancer Res..

[130] Farahani H., Alaee M., Amri J., Baghinia M.-R., Rafiee M. (2020). Serum and Saliva Concentrations of Biochemical Parameters in Men with Prostate Cancer and Benign Prostate Hyperplasia. Lab. Med..

[131] Abbasabad G.D., Khojasteh S.M.B., Naji H.E., Zamani M.R., Hajipour H., Serati-Nouri H. (2018). An Interleukin-6 Single Nucleotide Polymorphism and Susceptibility to Prostate Adenocarcinoma and Bone Metastasis in an Iranian Population. Asian Pac. J. Cancer Prev..

[132] Kelly R.S., Heiden M.V., Giovannucci E.L., Mucci L.A. (2016). Metabolomic Biomarkers of Prostate Cancer: Prediction, Diagnosis, Progression, Prognosis and Recurrence. Cancer Epidemiol. Biomark. Prev..

[133] Constâncio V., Nunes S.P., Moreira-Barbosa C., Freitas R., Oliveira J., Pousa I., Oliveira J., Soares M., Dias C.G., Dias T. (2019). Early Detection of the Major Male Cancer Types in Blood-Based Liquid Biopsies Using a DNA Methylation Panel. Clin. Epigenet..

[134] Kosaka T., Hongo H., Oya M. (2019). Complete Response with Early Introduction of Cabazitaxel in a Patient with Multiple Lung Metastases of Castration-Resistant Prostate Cancer Following the Early Detection of Metastases Using Liquid Biopsy: A Case Report. BMC Cancer.

[135] O’Reilly E., Tuzova A.V., Walsh A.L., Russell N.M., O’Brien O., Kelly S., Dhomhnallain O.N., DeBarra L., Dale C.M., Brugman R. (2019). EpiCaPture: A Urine DNA Methylation Test for Early Detection of Aggressive Prostate Cancer. JCO Precis. Oncol..

[136] Brikun I., Nusskern D., Decatus A., Harvey E., Li L., Freije D. (2018). A Panel of DNA Methylation Markers for the Detection of Prostate Cancer from FV and DRE Urine DNA. Clin. Epigenet..

[137] Markou A., Lazaridou M., Paraskevopoulos P., Chen S., Świerczewska M., Budna J., Kuske A., Gorges T.M., Joosse S.A., Kroneis T. (2018). Multiplex Gene Expression Profiling of In Vivo Isolated Circulating Tumor Cells in High-Risk Prostate Cancer Patients. Clin. Chem..

[138] Budna-Tukan J., Świerczewska M., Mazel M., Cieślikowski W.A., Ida A., Jankowiak A., Antczak A., Nowicki M., Pantel K., Azria D. (2019). Analysis of Circulating Tumor Cells in Patients with Non-Metastatic High-Risk Prostate Cancer before and after Radiotherapy Using Three Different Enumeration Assays. Cancers.

[139] Chen J., Cao S., Situ B., Zhong J., Hu Y., Li S., Huang J., Xu J., Wu S., Lin J. (2018). Metabolic Reprogramming-Based Characterization of Circulating Tumor Cells in Prostate Cancer. J. Exp. Clin. Cancer Res..

[140] Panigrahi G.K., Praharaj P.P., Kittaka H., Mridha A.R., Black O.M., Singh R., Mercer R., van Bokhoven A., Torkko K.C., Agarwal C. (2019). Exosome Proteomic Analyses Identify Inflammatory Phenotype and Novel Biomarkers in African American Prostate Cancer Patients. Cancer Med..

[141] Hofmann L., Sallinger K., Haudum C., Smolle M., Heitzer E., Moser T., Novy M., Gesson K., Kroneis T., Bauernhofer T. (2020). A Multi-Analyte Approach for Improved Sensitivity of Liquid Biopsies in Prostate Cancer. Cancers.

[142] Cheung P.K., Ma M.H., Tse H.F., Yeung K.F., Tsang H.F., Chu M.K.M., Kan C.M., Cho W.C.S., Ng L.B.W., Chan L.W.C. (2019). The Applications of Metabolomics in the Molecular Diagnostics of Cancer. Expert Rev. Mol. Diagn..

[143] Crigna A.T., Samec M., Koklesova L., Liskova A., Giordano F.A., Kubatka P., Golubnitschaja O. (2020). Cell-Free Nucleic Acid Patterns in Disease Prediction and Monitoring-Hype or Hope?. EPMA J..

[144] Hashimoto K., Nishimura S., Ito T., Oka N., Akagi M. (2021). Limitations and usefulness of biopsy techniques for the diagnosis of metastatic bone and soft tissue tumors. Ann. Med. Surg..

[145] Gerner C., Costigliola V., Golubnitschaja O. (2020). Multiomic Patterns in Body Fluids: Technological Challenge with a Great Potential to Implement the Advanced Paradigm of 3p Medicine. Mass Spectrom. Rev..

[146] Golubnitschaja O., Liskova A., Koklesova L., Samec M., Biringer K., Büsselberg D., Podbielska H., Kunin A.A., Evsevyeva M.E., Shapira N. (2021). Caution, “normal” BMI: Health risks associated with potentially masked individual underweight EPMA Position Paper 2021. EPMA J..

